# Simple regression models as a threshold for selecting AFLP loci with reduced error rates

**DOI:** 10.1186/1471-2105-13-268

**Published:** 2012-10-16

**Authors:** David L Price, Michael D Casler

**Affiliations:** 1 Department of Agronomy, University of Wisconsin-Madison, 1575 Linden Dr, Madison, WI 53706, USA; 2 USDA-ARS, U.S. Dairy Forage Research Center, 1925 Linden Dr, Madison, WI, 53706, USA

## Abstract

**Background:**

Amplified fragment length polymorphism is a popular DNA marker technique that has applications in multiple fields of study. Technological improvements and decreasing costs have dramatically increased the number of markers that can be generated in an amplified fragment length polymorphism experiment. As datasets increase in size, the number of genotyping errors also increases. Error within a DNA marker dataset can result in reduced statistical power, incorrect conclusions, and decreased reproducibility. It is essential that error within a dataset be recognized and reduced where possible, while still balancing the need for genomic diversity.

**Results:**

Using simple regression with a second-degree polynomial term, a model was fit to describe the relationship between locus-specific error rate and the frequency of present alleles. This model was then used to set a moving error rate threshold that varied based on the frequency of present alleles at a given locus. Loci with error rates greater than the threshold were removed from further analyses. This method of selecting loci is advantageous, as it accounts for differences in error rate between loci of varying frequencies of present alleles. An example using this method to select loci is demonstrated in an amplified fragment length polymorphism dataset generated from the North American prairie species big bluestem. Within this dataset the error rate was reduced from 12.5% to 8.8% by removal of loci with error rates greater than the defined threshold. By repeating the method on selected loci, the error rate was further reduced to 5.9%. This reduction in error resulted in a substantial increase in the amount of genetic variation attributable to regional and population variation.

**Conclusions:**

This paper demonstrates a logical and computationally simple method for selecting loci with a reduced error rate. In the context of a genetic diversity study, this method resulted in an increased ability to detect differences between populations. Further application of this locus selection method, in addition to error-reducing methodological precautions, will result in amplified fragment length polymorphism datasets with reduced error rates. This reduction in error rate should result in greater power to detect differences and increased reproducibility.

## Background

The ability to determine genotypes using molecular markers has provided a wealth of genetic information in numerous fields of study. In many biological fields genotype information is now critical in the decision making process. Due to the time and cost associated with these decisions, having accurate and reproducible data is essential. Technology improvements and reduced costs have resulted in genotype information increasing exponentially. As datasets grow larger it is inevitable that genotyping errors will occur [1]. Genotyping errors can be defined as a situation in which the observed genotype differs from the real genotype of an individual [2]. However, determination of the actual genotype of an individual is rarely possible and therefore genotyping errors are more often assayed by comparing genotypes obtained independently from the same individual. While genotyping error is understood to be a common occurrence in molecular genetic studies, few studies within the current literature document error rates associated with experiments [2]. The causes of genotyping errors can be numerous but are often associated with human error, scoring limitations, and biochemical anomalies [1]. As the number of samples and reactions increase, it can be expected that the number of erroneous genotypes within a dataset will also rise. Methods for controlling and identifying genotyping errors include standard experimental design procedures of randomization, replication, and proper controls [1-3]. As a result of these efforts genotyping error can be reduced, but is rarely eliminated within DNA marker datasets.

Amplified fragment length polymorphism (AFLP) is a popular dominant DNA marker technique that has been used for many different applications since its introduction nearly twenty years ago [4]. The technique is based on cleaving whole DNA using restriction enzymes, followed by PCR amplification of a subset of the cleaved fragments using selective primer combinations. Scoring of AFLP markers is subsequently based on the presence or absence of an amplified fragment. An AFLP locus is defined as a specific fragment size having either the present or absent allele. Applications of AFLP markers and their analysis have been thoroughly reviewed in the literature [5,6]. While next-generation sequencing has created a wealth of genetic information, the AFLP technique continues to provide useful information for numerous experimental questions. This continued use of the AFLP technique is largely due to its ability to screen a large number of genomically representative markers at a substantially lower cost compared to other techniques within non-model species with few genomic resources [6,7].

The introduction of AFLP fragment analysis using capillary electrophoresis has increased both the throughput and the quality of AFLP data [6]. This system has also increased the resolution at which fragments can be separated and therefore has resulted in a substantial increase in the number of fragments that can be used in analyses relative to gel electrophoresis. With the development of automated scoring software, fragments can now be compared at an ever-increasing resolution and error at the genotype calling stage can be greatly reduced [8]. Despite the error reduction at the genotype calling stage increased resolution may lead to the use of spurious fragments and other non-reproducible data. Both of these situations could contribute to an increased error rate within the dataset.

Within current AFLP literature, the existence of relatively high genotyping error is well documented [9,10]. Despite the high potential of genotyping error, reporting of error rate within AFLP datasets is rare. Being a dominant marker, only the presence or absence of a band is observable within AFLP datasets, therefore, error rate is estimated by calculating the ratio of observed differences between replicate samples and the total number of comparisons [2]. Use of all available loci in an analysis likely includes loci that have multiple erroneous alleles, possibly leading to incorrect conclusions or potentially increased amounts of noise within the dataset.

If error rate varies between loci, it is desirable to be able to identify those loci that have greater estimated amounts of error and remove those from the dataset. Previous studies have demonstrated inaccurate population substructure patterns in both datasets with high genotyping error rates and datasets using selected loci with very low error rates [7]. These results suggest that a tradeoff exists between reducing error rate and maintaining loci with high information content.

The objective of this paper is to outline a logical and computationally simple method for selecting loci that accounts for differences in error rate based on the frequency of present alleles at a given locus. It is hypothesized that by selecting loci using this method, error rate within an AFLP experiment can be reduced, thereby reducing the number of erroneous genotypes within a dataset while maintaining genomic diversity. This reduction in erroneous genotypes is expected to increase discrimination between differing samples and improve the ability to detect genetic differences of interest.

## Results

### Selection of loci with reduced error rate

Big bluestem is a warm season grass native to the North American prairie. From a geographically diverse panel of 458 big bluestem samples previously used in a study of genetic diversity, 81 samples were replicated and used in independent AFLP analyses [11]. Samples represented individual plants from 88 populations originating from three groups (Northeastern U.S.A., Wisconsin, and released accessions/cultivars). Nine *EcoRI*/*MseI* primer combinations were used for selective amplification resulting in 2711 polymorphic loci with a mean of 301.2 loci per primer combination. Locus-specific error rate ranged from 0% - 62%, although more than half of the loci had error rates less than 10% (Table 1). The estimated error rate per primer combination ranged from 9.6% - 13.3% with a mean of 12.5%. The number of present alleles per locus ranged from 0 to 154, with a mean frequency of 0.169.

**Table 1:** **Description of AFLP loci without selection**, **selected using Model 1**, **selected using Model 2 and selected using fixed error rate thresholds**

**Model**	**# of loci**	**Mean allele frequency**	**Error rate**
No Selection	2711	0.169	12.53%
Model 1	1173	0.181	8.83%
Model 2	417	0.159	5.93%
Fixed threshold 20%	2062	0.088	6.96%
Fixed threshold 10%	1457	0.046	3.91%
Fixed threshold 5%	915	0.025	1.99%

**Figure 1:**
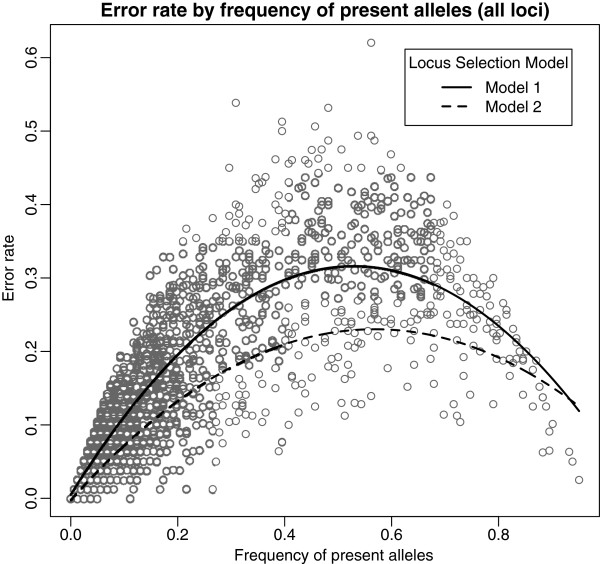
**Error rate by frequency of present alleles.** Scatterplot of locus-specific error rates in relation to the frequency of present alleles. Model 1 is polynomial model describing relationship (Y=0.0053+1.18X −1.11X^2^, p-value<0.001, R^2^=0.786). Model 2 describes relationship of points with error rates less than predicted by Model 1 (Y=−0.0020+0.82X −0.72X^2^, p-value<0.001, R^2^=0.844).

Simple regression using a second-degree polynomial term was used to model the relationship between error rate and the frequency of present alleles. The resulting model for this analysis was Y=0.0053+1.18X −1.11X^2^ (p-value<0.001, R^2^=0.786) (Figure 1). Using this model all loci with error rates greater than predicted by the model were removed from the analysis. A total of 1538 loci were removed using this procedure (57% removed). The removal of these loci resulted in a reduction of the mean error rate to 8.8%, a 29% reduction in error rate compared to using all loci. The mean frequency of present alleles increased slightly to 0.181.

Following the same procedures regression analysis was repeated on the 1173 loci selected under the first model. This analysis resulted in a second model predicting error rate from the frequency of present alleles, Y=−0.0020+0.82X −0.72X^2^ (p-value<0.001, R^2^=0.844) (Figure 1). Again, those loci with error rates greater than predicted by the model were removed from the analysis. Using this procedure 756 loci were removed, reducing the number of loci to 417 (64% removed). The mean error rate of the remaining loci was 5.9% and the mean frequency of present alleles was 0.159. Additional rounds of selection were not initiated due to the limited numbers of loci that would have been available after further rounds of selection.

For comparison, locus selection was also conducted using fixed thresholds of 20%, 10%, and 5%. Using these thresholds, all loci having error rates greater than the threshold were removed from analysis. Mean error rate was reduced dramatically, deceasing to less than 2% using the most conservative threshold (Table 1). While using a fixed error rate threshold reduced error and maintained a high number of loci, it came at the expense of a decrease in the mean allele frequency, reducing the mean from 0.169 per locus to 0.025 per locus. This reduction in mean allele frequency indicates that by using a fixed error rate threshold the dataset becomes dominated by loci with low allele frequencies.

### Testing

**Figure 2:**
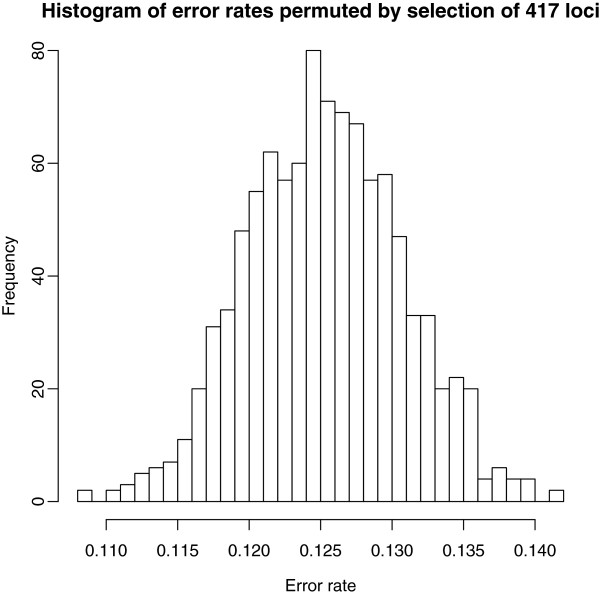
**Histogram of error rates permuted by selection of random loci.** Histogram of 1000 permuted error rates created by selection of 417 random loci. Mean of permuted error rates is 12.5%, compared to 5.9% of those loci selected using locus selection procedure.

Significance of the observed changes due to selection was tested by permutation test, randomly selecting 417 loci from the original 2711 loci. This procedure was repeated 1000 times, creating a distribution of error rate for randomly selected loci (Figure 2). The mean error rate of the permuted selections ranged from 10.8% - 14.2%, with a mean of 12.5%. Error rate of the loci selected using Model 2 was 5.9%, significantly different from the bounds of the permuted null distribution (p-value = 0.001).

### Implementation

Analysis of molecular variance analysis (AMOVA) was used to test the practical significance of the observed reduction in error rate. Three separate analyses were conducted on 458 big bluestem samples using different subsets of loci: (1) the original loci, (2) the loci selected under Model 1, and (3) the loci selected under Model 2. Samples represented individual plants from 88 populations originating from three geographic groups (Northeastern U.S.A, Wisconsin, and released accessions mostly from the Central U.S.A.) [11]. Variance was partitioned in a hierarchical manner among groups, among populations within groups, and plants within populations. Using loci selected under Model 1 AMOVA analysis demonstrated an increase in the variance explained among groups, increasing from 2.8% to 3.7% (Table 2). Using loci selected using Model 2 the percentage of variance explained by populations within groups increased from 6.6% to 9.6% and variance among groups increased from 2.8% to 4.2%. The observed changes in the amount of explainable variance demonstrate that the locus selection procedure has practical effects, in this example resulting in increased power to detect difference between groups and populations. Due to the geographic isolation of the populations tested in this example some amount of genetic divergence was expected. By removing more error prone loci from the dataset evidence of this divergence is more apparent.

**Table 2:** Analysis of molecular variance (AMOVA) for 458 individuals of big bluestem based on loci without selection, loci selected using Model 1, and loci selected using Model 2

	**No selection**		**Error less than Model 1**		**Error less than Model 2**	
**Source**	**Est. Variance**	**% of Variance**	**Est. Variance**	**% of Variance**	**Est. Variance**	**% of Variance**
Among groups	7.751	2.8%	4.448	3.7%	1.853	4.2%
Populations within groups	18.015	6.6%	7.937	6.5%	4.215	9.6%
Plants within populations	247.659	90.6%	108.887	89.8%	37.957	86.2%
Total	273.425	100.0%	121.271	100.0%	44.026	100.0%

All variances were significant (p-value <0.001) based on permutation test.

Using the fixed rate error thresholds, changes to the amount of variance explained within in the AMOVA analysis differed from those observed using the moving error rate threshold. Variance explained by populations within groups increased as the error rate threshold became stricter, from 6.6% to 9.1% (Table 3). In contrast, variance explained among groups decreased from 2.8% to 2.3% by using a more strict error rate threshold (Table 3). This decrease in the amount of explainable variance and the observed difference from the results using the moving error rate threshold may be due to the major reduction in moderate frequency alleles that resulted from using a fixed error rate threshold.

**Table 3:** Analysis of molecular variance (AMOVA) for 458 individuals of big bluestem based on loci selected using fixed error rate thresholds

	**20**% **error rate threshold**		**10**% **error rate threshold**		**5**% **error rate threshold**	
**Source**	**Est. Variance**	**% of Variance**	**Est. Variance**	**% of Variance**	**Est. Variance**	**% of Variance**
Among groups	3.923	2.8%	1.540	2.5%	0.538	2.3%
Populations within groups	10.657	7.7%	5.015	8.3%	2.110	9.1%
Plants within populations	123.658	89.5%	54.155	89.2%	20.669	88.7%
Total	138.237	100.0%	60.709	100.0%	23.316	100.0%

All variances were significant (p-value <0.001) based on permutation test.

## Discussion

Increasing numbers of AFLP loci require that discretion be used in selecting loci for further analysis. Ideally, selected loci should be reproducible and have as low of error rate as possible while maintaining genomic diversity. It is therefore essential that a method be in place for determining which loci best fit these requirements. The method proposed in this paper uses a simple regression approach to implement a moving error rate threshold that is optimized based on the frequency of present alleles at a given locus. By using the frequency of present alleles and simple regression models an error rate threshold can be set that is both computationally simple and accounts for the relationship between the frequency of present alleles and error within a given dataset. With the widespread use of the AFLP technique in various species and differing equipment with various protocols, a customizable error threshold accounts for technical marker variation that may be unique to an individual dataset.

The need for a moving error rate threshold can be demonstrated within the example dataset of replicated big bluestem AFLP markers. When a fixed error rate threshold (e.g. 10%) is used the majority of the selected loci are those with extremely high or low frequencies of present alleles. This effect is easily observed within a scatterplot showing the relationship between frequency of present alleles and error rate (Figure 1). Using the fixed rate threshold of 20% reduces the frequency present alleles by 48% to 0.088 (Table 1). This reduction is even more drastic if a threshold of 5% is used, reducing the frequency of present alleles to 0.025 (Table 1). Use of a fixed error rate threshold effectively eliminates loci with moderate frequencies of present alleles, therefore introducing bias into the selection process. It is important that these moderate frequency alleles be included, as those loci having high or low frequencies of present alleles only represent relatively rare alleles. In contrast, loci having a moderate frequency of present alleles represent common alleles. These common alleles are often those that are important for distinguishing differences between groups and populations of samples that differ largely in allele frequencies. If loci with moderate frequencies of present alleles are removed, the ability to distinguish between populations may be diminished.

The effectiveness of the proposed locus selection method for reducing error rate was demonstrated using an AFLP dataset resulting from experiments to test the genetic diversity of the prairie species big bluestem. By using a simple regression model with a second-degree polynomial term to set the error rate threshold, error rate within the dataset was reduced from 12.5% to 8.8%. Applying the same technique to selected loci resulted in an additional decrease in error rate to 5.9%. Overall the use of the proposed methods reduced error rate by more than one half. Prior to locus selection this dataset had an error rate that would have been considered relatively high. By using Model 2 to select loci, the error rate was lowered to a level that is within the range typically found in an AFLP datasets [8-10]. Comparatively, the use of fixed rate error thresholds was very effective at reducing error rate, reducing error rate to 7.0%, 3.9%, and 2.0% for respectively for thresholds of 20%, 10%, and 5% (Table 1). Despite these large decreases in mean error rate, one must consider the tradeoff that is made between reducing error rate and maintaining genomic diversity. The use of fixed error rate thresholds dramatically decreased the mean frequency of present alleles compared to the use of a moving error rate threshold in model 1 and model 2 (Table 1). This reduction indicates that the majority of loci conserved using fixed error rate thresholds have very low frequencies of present alleles. By using theses loci in further analyses much of the genomic diversity that exists within the dataset is lost. Previous studies have demonstrated that larger datasets having higher error rate can yield greater information than a small number of loci with little error [7]. This reduction in information content may be related to the reduction of loci with moderate allele frequencies, as a result of using fixed error rate thresholds.

Equally important to lowering error rate to acceptable levels is the effect that reduced error can have on the ability to eliminate noise in the dataset and increase the ability to detect differences between samples. In the example shown in this paper, locus selection resulted in increased differentiation between samples from differing groups under Model 1. Using Model 2 the ability to differentiate between samples from differing populations within groups also increased. In contrast, the use of fixed rate error thresholds decreased the amount of observable differentiation among groups to levels lower than observed without locus selection. In this example the differences in differentiation observed using the moving error rate thresholds made a major contribution to the understanding of big bluestem diversity in the tested samples [11]. It can be expected that results of a similar magnitude will be observed in other AFLP datasets.

## Conclusions

High error rates within AFLP datasets can cause increased noise and possibly incorrect conclusions. Using an arbitrary error rate threshold to remove loci with high error rates can bias locus selection by removing moderate frequency alleles. This paper demonstrates the use of simple regression to model the relationship between error rate and the frequency of present alleles. These models create moving error rate thresholds that can be subsequently used for selecting loci for use in future analyses with reduced error rates. In the present example, using loci selected with the proposed method resulted in a reduction in mean error rate for AFLP markers in big bluestem. In addition to reducing error, the removal of loci with high error rates from the dataset increased differentiation between samples from differing groups and populations.

Genotyping errors within AFLP datasets have been shown to be nontrivial. Despite this, error rates for AFLP datasets are rarely measured or reported. The use of a locus selection procedure such as those proposed carries with it an inherent tradeoff between reducing error rate and maintaining genomic diversity. This tradeoff must be balanced as the reduction of genomic diversity resulting from arbitrary or strict locus selection may introduce bias in a dataset by removing informative alleles. Equally, the inclusion of substantial genotyping error may reduce power to detect differences within a dataset and will reduce repeatability. Within the example dataset in this paper two rounds of selection were considered appropriate due to the large number of loci, high initial error rate, and the goals for this data. Within other datasets the number of rounds of locus selection may need to change in order to meet the objectives of an individual study and the mean error rate that is acceptable for a given study. The proposed methods provide a tool that researchers can use to better understand genotyping error within AFLP datasets, leading to improved reproducibility and greater ability to discern genetic differences.

## Methods

Plant materials for this study came from a diverse collection of natural and cultivated populations of big bluestem (Andropogon gerardii Vitam.) [11]. A single leaf was collected from 458 plants associated with 88 populations originating from three geographic groups (Northeastern U.S.A., Wisconsin, and released accessions mostly from the Central U.S.A.). DNA was extracted from lyophilized leaf tissue using a modified sorbitol extraction method [12]. Of the 458 samples 81 (17.6%) were randomly selected as replicates and an independent DNA extraction was performed for all replicates. Following extraction, DNA concentration was normalized to 100 ng uL-1. DNA samples were distributed randomly into 96-well plates in order to minimize plate to plate variation [6]. Multiple negative controls containing only water were distributed randomly in each plate to control for failed amplifications.

AFLP amplification was completed using the protocol of Clarke and Meudt [13]. Nine selective primer combinations were used by creating all combinations of three *MseI* selective primers (*MseI*+CGA, *MseI*+CTG, and *MseI*+CTT) and three *EcoRI* selective primers (*EcoRI*+AAG, *EcoRI*+AGC, and *EcoRI*+AGG, fluorescently labeled with FAM, TAMRA and HEX respectively). Following selective amplification products were combined by *MseI* primer, such that multiple primer combinations could be analyzed in a single run. Amplified fragments were separated using ABI 3730 automatic capillary DNA sequencer (Applied Biosystems, Foster City, CA) and electropherograms were processed using Peak Scanner v1.0 (Applied Biosystems) using default parameters. Fragment sizes were determined relative to a GeneScan 500 ROX Size Standard (Applied Biosystems). Fragment data was scored for presence (1) or absence (0) using the RawGeno package v1.1-2 [8] in the R statistical package ( http://www.r-project.org) [14]. Scoring parameters used were as follows: Scoring range 100–400 bp; minimum intensity, 100 rfu; minimum bin width, 0 bp; maximum bin width, 2 bp. Closely sized bins were removed. Bins with rare alleles (less than 3 present alleles) were removed from further analysis. All monomorphic bands were removed from subsequent analyses. Samples having similar banding patterns to the negative controls were removed from the analysis.

Fragment scoring results were used to create a binary matrix table indicating the presence or absence of a fragment at each locus. Results from the replicated samples were compared to their replicate, and it was determined if the presence or absence of a fragment matched between replicates. For each locus a locus-specific error rate was estimated by dividing the number of mismatches by the total number of comparisons [2]. A mean error rate was subsequently calculated by dividing the sum of the estimated error for all loci by the total number of loci.

Locus-specific error rates were compared to the frequency of individuals having the present allele phenotype for a given locus. This comparison was visualized with a scatterplot displaying the frequency of present alleles on the x-axis and locus-specific error rate on the y-axis. The relationship was quantified by creating a model using simple regression analysis with a second-degree polynomial term in R [14] to predict error rate as a function of frequency of present alleles. Locus selection was done by removing all loci having error rates greater than predicted by the frequency of present alleles based on the predictive model (Model 1). Following the initial selection, a second regression analysis was completed on the selected loci following the same procedure as previously described. The second model (Model 2) was used to perform an additional round of locus selection, removing those loci with greater than expected error rates based on the second model. Mean error rates were subsequently estimated for those loci selected in both the initial and the second selection.

To test the significance of the change in error rate due to the selection procedure, a permutation test was conducted using the complete set of 2711 loci. A custom Macro was created using MS visual basic (Microsoft Corporation, Redmond, WA), to randomly select a defined number of loci. Following selection the mean error rate for the selected loci was estimated using the previously outlined methods. The random selection and estimation procedure was repeated 1000 times, and a distribution of mean error rates was created. The hypothesis that the error rates from the loci selected based on the models were significantly different than those selected randomly was tested by comparing the estimated error rate of those loci selected under each model to the distribution of those selected randomly. The p-value of each being equal to the proportion of those means selected randomly with equal or lower error rates than those selected under the models.

Fixed rate error thresholds were implemented by removing all loci with error rates greater than 20%, 10%, or 5%. Mean error rates were subsequently estimated for those loci selected at each threshold.

Comparisons of the effects of locus selection on analysis results were done using the data without locus selection, loci selected under Model 1, loci selected under Model 2, and loci selected using fixed thresholds. The analysis of molecular variance test [15] was performed on all 458 samples using GenAlEx 6 [16]. Variance was partitioned in a hierarchal manner among and within the three groups of samples, collections sites or accession, and individual samples. Significance of the AMOVA was tested using 999 permutations.

## Competing interests

The authors declare no competing issues related to methods described within in this manuscript.

## Authors’ contributions

DLP developed the methodology, conducted the experimental analysis, and was the primary author of the manuscript. MDC assisted in the design, analysis, and review of the manuscript. Both authors read and approved the final version of the manuscript.
